# The investigation of combined ventilation-biofilter systems using recycled treated wastewater on odor reduction efficiency

**DOI:** 10.5713/ajas.19.0777

**Published:** 2019-12-24

**Authors:** Andi Febrisiantosa, Hong L. Choi, Anriansyah Renggaman, Sartika I. A. Sudiarto, Joonhee Lee

**Affiliations:** 1 Department of Agricultural Biotechnology, Research Institute for Agricultural and Life Science, Seoul National University, Seoul 08826, Korea; 2 Research Unit for Natural Product Technology, Indonesian Institute of Sciences, Yogyakarta 55861, Indonesia; 3 Resourcification Research Center for Crop-Animal Farming, Seoul 08800, Korea; 4 School of Life Science and Technology, Institute Teknologi Bandung 40132, Indonesia; 5 Institute of Livestock Environmental Management, Daejeon 34065, Korea

**Keywords:** Ammonia, Odor Reduction, Recycling Slurry System, Ventilation, Biofilter, Pig House

## Abstract

**Objective:**

The present study aimed to evaluate the performance of odor abatement by using two different ventilation-biofilter systems with recycled stablized swine wastewater.

**Methods:**

The performance of odor removal efficiency was evaluated using two different ventilation-biofilter-recycled wastewater arrangements. A recirculating air-flow ventilation system connected to a vertical biofilter (M1) and a plug-flow ventilation system connected to a horizontal biofilter (M2) were installed. Water dripping over the surface of the biofilter was recycled at a flow rate of 0.83 L/h in summer and 0.58 L/h in winter to reduce odorous compounds and particulate matter (PM). The experiments were performed for 64 days with M1 and M2 to investigate how these two ventilation-biofilter systems influenced the reduction of odor compounds in the model houses. Odorous compounds, NH_3_ and volatile organic compounds (VOCs) were analyzed, and microclimatic variables such as temperature, humidity, and PM were monitored.

**Results:**

Ammonia concentration inside M1 was about 41% higher on average than that in M2. PM and total suspended particles (TSPs) inside M1 were about 62.2% and 69.9%, respectively, higher than those in M2. TSPs in the model house were positively correlated with the concentration of NH_3_ and VOCs.

**Conclusion:**

M2 emitted lower concentration of odorous compounds than M1. Moreover, M2 could maintain the optimum temperature condition for a swine house during the cooler season. The plug-flow ventilation–horizontal biofilter system could be used for pig houses to minimize air pollution produced by swine farming activities and maintain optimum microclimate conditions for pigs.

## INTRODUCTION

Intensive confined livestock operations have been remarkably increased over the past few decades to meet the high demand for meat products [1,2]. However, the increase in the number and size of confinement facilities has caused air pollution problems. In swine farms, the ventilated air exhausted from an animal house may contain high concentrations of odorous gases and particulate matter (PM). The emission of odors and volatile organic compounds (VOCs) from livestock buildings are representative of air pollution problems and have become a crucial public health concern [3]. VOCs contribute to the formation of photochemical smog, ozone, and respirable suspended particulates in the atmosphere [4]. PM from pig houses contains microorganisms and bioaerosols that increase the prevalence of respiratory infections in people living in neighboring areas [5].

About 50% of odor emission from livestock production is contributed by livestock buildings (30%) and manure storage facilities (20%) [6]. Gases such as NH_3_, CO_2_, CH_4_, and sulfur compounds (hydrogen sulfide and mercaptans) are the most abundantly produced by piggeries [7]. The importance of managing odorous compounds, VOCs, and PM from livestock facilities has recently been increasingly acknowledged. Adverse environmental problems caused by the swine industry need to be reduced by developing effective methods for handling a wide range of airborne compounds.

Various air pollution control technologies have been developed such as activated carbon adsorption, wet scrubbing, and masking agents [8]. Unlike other physicochemical technologies, biofiltration has been recognized as a reliable technology to control low and moderate concentrations of waste gases containing odors and VOCs [9,10]. Biofilters are used to reduce odor emissions from livestock facilities located in residential areas and are effective in reducing odor loads by about 40% to 83% [11]. Biofilters need adequate air contact time to reduce air pollutants effectively [12]. In other words, the ventilation rate of livestock facilities can play an essential role in the effective operation of biofilters. Zong et al [13] investigated the application of different ventilation systems to identify cost-effective methods for the purification of exhaust air from pig buildings.

However, studies on swine housing that incorporates a ventilation-biofilter system to reduce odor in pit swine houses are rare. Therefore, this study aimed to assess the effects of two ventilation-biofilter systems (i.e., M1, a recirculating air-flow ventilation system connected to a vertical biofilter; M2, a plug-flow ventilation system connected to a horizontal biofilter) on odor emission and microclimate conditions with the same recycling of dripping water by using the biofilter.

## MATERIALS AND METHODS

### Experimental pig house

**Figure 1:**
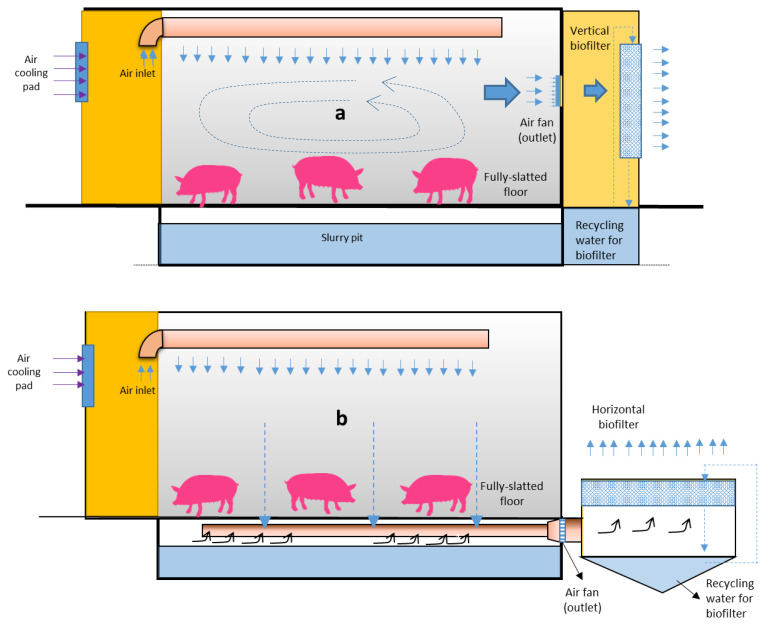
The experimental pig house (a) M1 (recirculation flow–vertical biofilter); (b) M2 (plug-flow–horizontal biofilter)

Two trials were conducted in experimental model pig houses that were designed to facilitate the assessment of various biofilters incorporated with ventilation systems. The experiment was conducted for 64 days from October 10 to December 13, 2017. Each model pig house had 9 fattening pigs in an area of 12 m^2^ (3 m wide×4 m long). The pig housing density was 1.3 m^2^/head, which is greater than that in the field (0.8 to 1.0 m^2^/head). Two ventilation systems connected to two types of biofilter systems were investigated, as shown in Figure 1a and 1b. The biofilter pads were made of cellulose materials. The dripping treated wastewater over the surface of the biofilters was recycled at a flow rate of 0.83 L/h in summer and 0.58 L/h in winter to reduce odorous compounds and PM by moisturizing the biofilter pads during the operation. Pigs were fed *ad libitum* (free ration). They also had unrestricted access to water. Ratios of crude proteins contained in the food intake were 16% and 14.5% during the growing and finishing periods, respectively. The floor of the piggery was fully slatted for slurry storage (pit) underneath.

In M1 (Figure 1a), air enters through the inlet duct, flows downward, recirculates above the slatted floor in the compartment, and exhausts out through the vertical biofilter. In M2 (Figure 1b), air enters through the inlet duct, flows downward into the pit, and is exhausted out through the horizontal filter. The flow rate was controlled using exhaust fans to maintain the temperature in the building constant (23°C) irrespective of the external weather conditions, with the highest flow at 0.6 m^3^/s [7]. Ventilation rates through the exhaust channels were automatically controlled according to the set compartment temperature (Vent System A/S; Roslev, Denmark).

The dripped water for M1 was stored in a reservoir (2.8 m ×1.5 m×1.0 m in depth) for recycling. The vertical biofilter was assembled from corrugated plastic sheets. The basic configuration of the horizontal biofilter for M2 was the same as M1 except that the treated wastewater was sprayed over the horizontal pads.

### Analysis of microclimatic variables and odorous compounds

Indoor (compartment and pit) and outdoor air temperature and humidity were recorded. A hygrothermograph (SK-110TRH; SATO, Tokyo, Japan) was used to measure air temperature and relative humidity (RH). PM was analyzed using an aerosol mass monitor (GT-331; SIBATA, Soca-city, Japan), and the mass concentrations of PM10, PM7, and PM1 (average aerodynamic diameter: 10, 7, and 1 μm, respectively) and of total suspended particles (TSPs) were obtained simultaneously, at a flow rate of 2.83 L/min.

In all, 11 types of VOCs were analyzed in this study, according to the method described by Kumari et al [14]. These included two sulfuric compounds (dimethyl sulfide [DMS] and dimethyl disulfide [DMDS]); six odorous volatile fatty acids (VFAs; acetic acid [AA], propionic acid [PA], butyric acid [BA], isobutyric acid, valeric acid [VA], and isovaleric acid); two indoles (indole and skatole); and one phenol (*p*-cresol). The air was sampled twice a day (11:00 and 16:00) for 5 min by using a 1 L Tedlar bag (No. 22053; Restek, Bellefonte, PA, USA), and an average for the day was obtained. The collected air samples were analyzed within 24 h by using gas chromatography/mass spectrometry (GC/MS; Agilent GC6890N/5975C; Santa Clara, CA, USA) in the laboratory. A Gastec probe (Gastec Co., Ltd., Kanagawa, Japan) was used to measure the concentrations of NH_3_.

The differences in NH_3_ concentration across the biofilters were used to calculate the biofilter NH_3_ removal efficiency (RE), as described by Ashtari et al [15]. Equation (1) was used to calculate the NH_3_ RE by using the inlet (C_1_, ppm) and exhaust (C_2_, ppm) NH_3_ concentrations.

Equation (1) $$RE=\left(\frac{C_{1}-C_{2}}{C_{1}}\right)\times 100$$

### Statistical analysis

Statistical evaluations were performed using SPSS ver. 22 (IBM SPSS Inc., Chicago, IL, USA). Each variable was first compared using analysis of variance at a 5% significance level. The average values of the quantitative factors under evaluation were compared using Tukey’s test.

## RESULTS AND DISCUSSION

### Microclimate condition and NH_3_ concentration

**Figure 2:**
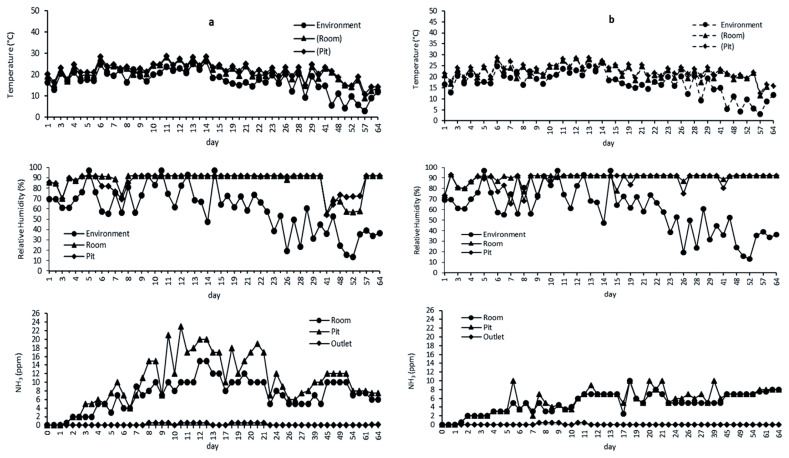
The average temperature, relative humidity (RH), and ammonia (NH_3_) concentration in (a) M1 and (b) M2 during the experiment.

Temperature, RH, and NH_3_ concentration in M1 and M2 during the experiment are shown in Figure 2a and Figure 2b, respectively. M1 and M2 had a similar temperature variation from late summer to winter. However, M2 showed better temperature maintenance during the cooler season than M1. Temperatures were generally lower in M2 than in M1, even though the inside compartment temperature was set at 23°C to maintain a proper temperature range for the pigs. M2 showed better maintenance of the inside temperature during the cooler season at the minimum ventilation rate of 0.6 m^3^/s, as the envelop was insulated with urethane foam in M2, whereas it was insulated with styrofoam in M1. The temperature in the pit was found to be mostly the same in both M1 and M2.

The inside RH of M1 and M2 showed different patterns during the experimental period (late summer to early winter). Humidity in M2 was higher than that in M1 in warmer weather. The recirculating ventilation system was better in maintaining lower inside humidity for pig growth; however, no difference in RH was noted between M1 and M2 in the cooler weather. The observed RH reached 90%, which was higher than the optimal range of RH for pig growth (reported ideal range of 50% to 70%) [16], and the difference in RH was greater between the environment and the compartment. The air passing the evaporative cooling unit absorbs water, and hence lowers the air temperature before entering the pig house. In addition, water vapor physiologically produced by the pigs increases RH inside the pig house [17].

The variation in NH_3_ concentration during the experiment is shown in Figure 2. In M1, the NH_3_ concentration in the pit was higher than that of the aerial space over the bedding floor in the model pig house. The mean NH_3_ in M1 was 7.09, 10.27, and 0.15 ppm, whereas that in M2 was 4.93, 5.56, and 0.06 ppm in the aerial space, pit, and at the outlet, respectively. The NH_3_ concentration in the aerial space of M1 was higher by about 42% than that of M2; thus, different ventilation systems had different NH_3_ emission rates. The higher NH_3_ concentration in M1 may be attributed to the recirculating flow pattern in the aerial space induced by the ventilation system. Zong et al [18] revealed a significant turbulence flow in the system with recirculating ventilation. Furthermore, a partial plug-flow ventilation system has been shown to have the ability to improve the indoor air quality remarkably and reduce NH_3_ emission, if combined with air purification systems. The NH_3_ concentration from both M1 and M2 decreased remarkably to a level below 0.5 ppm after passing the biofilters. The greater amount of NH_3_ reduction across the biofilter in M1 and M2 was mainly attributed to solubility by dripping water. In addition, NH_3_ was not emitted during the biofiltration operation using recycled treated wastewater. The average pH of dripping water recorded 6.89 (6.65 to 7.38) in M1, and 5.83 in M2 (5.15 to 7.07) respectively. The nitrogen in M1 and M2 in the organic compound is oxidized but is not converted to ammonia at pH below 7.0 [19].

### Effect on volatile organic compounds and particulate matter

Although all VOCs were analyzed, VA, *p*-cresol, and skatole were not detected (Table 1). Among the VFA compounds, PA and BA showed remarkably different levels between M1 and M2. Initial PA and BA concentrations in the aerial space in M2 were 8.6 ppbv and 3.9 ppbv, respectively, which were significantly higher (p<0.05) than those in M1 (4.6 ppbv and 0.8 ppbv, respectively). After the biofilter system was installed, the PA level between M1 and M2 was not significantly different (p>0.05), whereas BA level was higher (p<0.05) at the outlet of M1 (5.2 ppbv) than at the outlet of M2 (1.6 ppbv).

VFAs such as AA, iso-butyric acid, iso-valeric acid, and indole remained almost at the same level before and after biofiltration. In fact, AA is soluble in water, whereas PA and BA are more soluble than in DMS. However, the levels of these fatty acids (AA, PA, and BA) were not reduced after the installation of the biofilters. This could be because the concentration of these VFAs is in equilibrium with that in the air space in the enclosed biofilter compartment. The biofiltration performed using recycled treated wastewater may not be effective in reducing most VFAs and indole. This could be because the odorous organic compounds may be dissolved increasingly in the recycled dripping water.

The initial average DMDS and DMS concentrations were not significantly different (p>0.05) between M1 (6.7 ppbv) and M2 (6.4 ppbv). However, after the installation of the biofilter systems, the DMS level in M2 (3.3 ppbv) was significantly higher than that in M1 (0.3 ppbv; p<0.05). The DMDS concentration was not higher than the DMS concentration and remained almost the same before and after biofiltration. Limited oxygen concentration may cause biological reactions that produce sulfuric odor compounds during the storage of swine wastewater in the pit.

M2 showed significantly lower (p<0.05) PM10 (122.8 μg/m^3^) and PM7 (68.3 μg/m^3^) than in M1 (PM10, 203.4 μg/m^3^ and PM7, 114.3 μg/m^3^; Table 1). However, after the biofilters were installed, the density of smaller PM (PM2.5, PM1) at the outlet of M1 and M2 was not significantly different (p>0.05). The level of TSPs in M2 (260.9 μg/m^3^) was significantly lower (p<0.05) than that in M1 (443.3 μg/m^3^). Unlike the plug-flow pattern in M2, the recirculating-flow pattern in M1 increased the density of PM and TSPs, which could be caused by the high turbulence intensity, as explained by Zong et al [18]. Flow patterns of ventilation systems have been shown to influence the density of PM and TSPs in a model pig house, as the nature of airflow is basically a vector that has the momentum to carry scalars such as PM, temperature, and humidity.

The Pearson correlation values of odorous compounds and microenvironment variables in the present study are shown in Table 2. In our study, NH_3_ was significantly correlated with humidity (r = 0.55), PM10 (r = 0.54), PM7 (r = 0.50), and TSP (r = 0.56). This could be because NH_3_ is dissolved with water vapor and is adsorbed with large-sized PMs than with smaller dust. DMS showed significant positive correlation with humidity (r = 0.59), PM10 (r = 0.66), PM7 (r = 0.55), and TSPs (r = 0.63), and DMDS showed positive correlation with temperature (r = 0.51).

DMS showed the same correlation pattern with hydrophilic NH_3_. This could be explained by the adsorption of these two compounds on organic PM. Iso-butyric acid was positive correlated with temperature (r = 0.59), and BA was negatively correlated with PM10 (r = −0.41).

### Biofilter condition and performance

**Figure 3:**
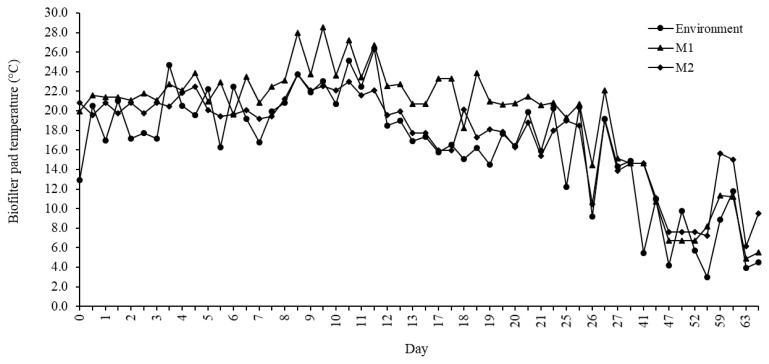
Biofilter pad temperature of the cross-ventilation–vertical biofilter (M1) and pit ventilation–horizontal biofilter (M2) during the experiment.

The biofilter pad temperature of the M1 and M2 systems during the experiment is presented in Figure 3. It gradually decreased with a decrease in environmental temperature. In warmer environmental conditions above 14°C, the temperature of the biofilter pad in M1 was higher than that in M2. Under cooler environmental conditions below 14°C, the temperature of the biofilter pad in M1 was lower than that of M2. This indicates that the horizontal biofilter can better respond to changes in ambient temperature and is suitable for microbial growth.

**Figure 4:**
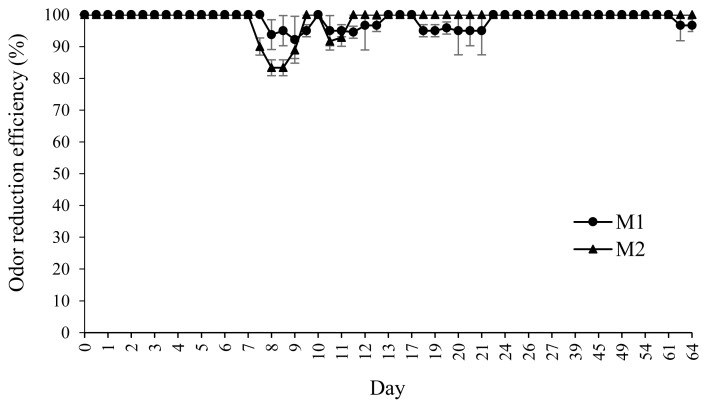
Average ammonia reduction efficiency of the biofilters in the recirculation flow–vertical biofilter (M1) and plug-flow–horizontal biofilter (M2) during the experiment. Values are expressed as mean±standard deviation.

The average NH_3_ reduction efficiency for M1 and M2 biofilters is shown in Figure 4. The NH_3_ reduction efficiency of M1 and M2 was not significantly different. The average NH_3_ reduction of M1 and M2 was 98.45% and 98.68%, respectively. According to this study, the different biofilters (orientation and pad materials) did not have a significant effect (p>0.05) on the NH_3_ reduction efficiency. Until the end of the experiment, the performance of both the biofilter systems remained above 95% on average. Thus, the biofilter does not need to be regenerated for every production period.

## CONCLUSION

The odor RE of two different systems using the combination of ventilation-biofilter-recycled wastewater arrangements was evaluated. A recirculating air-flow ventilation system connected to a vertical biofilter (M1) and a plug-flow ventilation system connected to a horizontal biofilter (M2) were installed.

The NH_3_ concentration in the pit was higher than that of the aerial space over the plastic perforated bedding floor in the model pig house in M1. The NH_3_ concentration in the aerial space of M1 was higher by about 42% than that of M2. This could be attributed to the recirculating flow pattern in the aerial space induced by the ventilation system. A plug-flow ventilation system has been shown to have the ability to remarkably improve indoor air quality and reduce NH_3_ emission if combined with biofilter systems. The NH_3_ concentration from both M1 and M2 was significantly decreased to a level below 0.5 ppm after passing the biofilters. This could be because NH_3_ is dissolved with water vapor and is adsorbed with large-sized PMs than by smaller dust. DMS showed significantly positive correlation with humidity and larger PM and TSPs, as well as with NH_3_. This could be because of the adsorption of these two compounds on organic PM.

Regarding VOC analysis, VA, *p*-cresol, and skatole were not detected at the initial phase. VFAs such as AA, iso-butyric acid, iso-valeric acid, and indole and DMDS remained almost at the same level before and after biofiltration. This may mainly be because the dripping treated wastewater was repeatedly recycled during the experiment; hence, the odorous organic compounds sedimented in the recycled water. The concentration of these VFAs was in equilibrium with that of the VFAs in the crossing airflow containing odorous organic compounds in the enclosed biofilter compartment. The biofiltration performed using recycled treated wastewater may not be effective in reducing most VOCs, upon the environment which the biofilters are confronted.

## Figures and Tables

**Table 1:** The odorous compounds and particulate matter in M1 and M2

Parameter	M1		M2	
	
	Inside	Outlet	Inside	Outlet
Odor compounds (ppbv)	------------------------------------------------------------------- ppbv -------------------------------------------------------------------------------			
Acetic acid	31.8±11.2	29.2±9.3	31.7±11.7	30.9±5.3
Propionic acid	4.6±1.3^a^	5.2±1.9^ab^	8.6±3.3^b^	8.0±3.2^ab^
Isobutyric acid	1.4±0.3	1.6±0.3	1.5±0.2	1.3±0.2
Butyric acid	0.8±0.3^a^	5.2±0.6^c^	3.9±1.4^b^	1.6±0.3^a^
Isovaleric acid	3.6±0.3	3.6±0.6	3.6±0.4	3.6±0.4
Valeric acid	ND	ND	ND	ND
P-Cresol	ND	ND	ND	ND
Indole	1.6±0.1	2.2±0.9	1.7±0.3	1.6±0.4
Skatole	ND	ND	ND	ND
Dimethyl sulfide	6.7±2.5^c^	0.3±0.1^a^	6.4±1.2^c^	3.3±1.2^b^
Dimethyl disulfide	0.4±0.1^ab^	0.3±0.0^a^	0.6±0.3^b^	0.5±0.2^b^
Particulate matter (PM)	------------------------------------------------------------------ μg/m^3^ --------------------------------------------------------------------------------			
PM10	203.1±30.4^c^	33.0±25.0^a^	122.8.0±36.8^b^	32.5±7.6^a^
PM7	114.3±41.0^c^	25.0±12.9^a^	68.3±15.3^b^	26.1±11.4^a^
PM2.5	13.7±2.7	9.3±5.4	11.7±1.8	10.4±7.2
PM1	6.5±2.1	5.6±3.2	5.3±1.2	6.1±4.5
Total suspended particle	443.3±79.7^c^	59.2±45.1^a^	260.9±98.3^b^	54.6±21.8^a^

Values are expressed as mean±standard deviation.

M1, a recirculating air-flow ventilation system connected to a vertical biofilter; M2, a plug-flow ventilation system connected to a horizontal biofilter; ND, not detected.

a–c Data with different letters in the same row are significantly different (p<0.05).

**Table 2:** Pearson correlation of measured odorants and microenvironment variables

Pearson correlation	Temperature	Humidity	PM10	PM7	PM2.5	PM1	TSP
Ammonia	0.40	0.55**	0.54**	0.50*	0.18	0.21	0.56**
AA	−0.06	0.15	−0.16	−0.21	−0.06	0.10	−0.16
PA	0.23	−0.22	−0.35	−0.34	0.07	0.15	−0.34
iBA	0.59**	−0.20	0.03	−0.08	0.00	0.04	0.10
BA	0.05	−0.11	−0.41*	−0.39	−0.09	−0.01	−0.39
iVA	0.13	−0.02	0.14	0.16	0.07	0.09	0.15
Indole	0.15	0.09	−0.14	−0.07	0.26	0.39	−0.17
DMS	0.32	0.59**	0.66**	0.55**	0.28	0.09	0.63**
DMDS	0.51*	0.10	0.31	0.28	0.24	0.28	0.30

PM, particulate matter; TSP, total suspended particle; AA, acetic acid; PA, propionic acid; *i*BA, isobutyric acid; BA, butyric acid; *i*VA, isovaleric acid; VA, valeric acid; DMS, dimethyl sulfide; DMDS, dimethyl disulfide.

* Correlation is significant at the 0.01 level.

** Correlation is significant at the 0.05 level.
